# Investigation of the Indentation Resistance of Aluminum Foam Sandwich Panels with Metallurgical Bonding Interfaces under Low-Velocity Impact

**DOI:** 10.3390/ma16062221

**Published:** 2023-03-10

**Authors:** Qiang Gao, Xixi Su, Peng Huang, Xi Sun, Zhanhao Feng, Guoyin Zu

**Affiliations:** School of Materials Science and Engineering, Northeastern University, Shenyang 110819, China

**Keywords:** metallurgical bonding interface, low-velocity impact, energy absorption, energy absorption efficiency

## Abstract

The impact resistance of aluminum foam sandwich panels (AFS) with metallurgical bonding interfaces prepared by the powder cladding rolling method was investigated. Low-velocity impact tests were conducted by using a drop-weight impact facility to explore the dynamic mechanical behavior, deformation and damage mechanisms, and energy absorption of AFS with metallurgical bonding interfaces. The effects of variation of impact energy, panel thickness, and specimen density on the energy absorption performance of AFS were quantitatively evaluated by energy absorption indicators. The results indicate that the load-displacement curve illustrates prominent three-stage characteristics when the impact energy is 120 J containing the front panel yielding stage, the foam core’s compressive and shear failure stage, and the back panel fracture stage. The impact strength of the sandwich structure increases with increasing panel thickness and specimen density. The AFS with metallurgical bonding interfaces presents favorable energy absorption efficiency under low velocity.

## 1. Introduction

The aluminum foam sandwich panel is a structural and functional integrated composite material with low density, high specific stiffness and strength, and outstanding energy-absorbing capability [1,2]. With excellent properties, AFS is widely used as lightweight energy-absorbing components which can enhance the impact resistance of structures, such as improving the crashworthiness of motor vehicles and explosion-proof facilities [3,4].

AFS are fabricated by combining porous aluminum foam as a flexible core layer with two layers of high stiffness/strength panels, solving the problem of low strength of the single aluminum foam. To a considerable extent, the mechanical properties of AFS depend on the interfacial bonding strength [5]. The present studies usually use an epoxy resin agent to compound the panel with the foam core. However, the resin adhesive layer is easy to deteriorate and delaminate under high temperatures or a corrosive environment, which seriously restricts the application of AFS [6,7]. Therefore, a few scholars have investigated metallurgical bonding formed by the diffusion of alloying elements between the panel and the foam core at high temperatures or pressure. Currently, there are very few researchers who can successfully prepare AFS by metallurgical bonding method, and therefore, metallurgical bonding is rarely employed. Yoshihiko et al. [8] and Peng et al. [9] has successfully prepared aluminum foam sandwich panels with excellent metallurgical bonding interfaces by stir friction welding. Yao et al. [10] prepared AFS with metallurgical bonding interfaces using liquid diffusion welding finding the AFS with metallurgical bonding had higher peel strength and fatigue life than those prepared by the adhesive process. However, the smaller size and higher cost make the above research difficult to achieve in industrial applications. With the increasing demand for highly stable interfaces and large-size sandwich structures, Zu et al. [11,12] developed the powder cladding rolling technology, which is proven to meet the needs of large-size products for industrial applications. The AFS with metallurgical bonding interfaces possesses high interfacial stability and interfacial bonding strength of the panel/foam core [13]. In addition, the AFS precursors can also be molded into complex parts before foaming, which is usually difficult to achieve through the adhesive bonding process [14].

AFS is usually used as an impact protection material to absorb large amounts of impact energy to protect the internal structure. As a result, they are inevitably subjected to collisions with external objects of various shapes and subjected to impact loads to produce local indentation deformation. For instance, Aerospace vehicles may be hit by debris and bird collisions during takeoff and landing [15,16]. Furthermore, the materials are hit by tools or debris falling during maintenance and service [17]. The structural damage evolution process and energy absorption mechanism were very complex because macroscopic local indentation deformation involves the cell’s compression, bending, and shearing. Therefore, it is important to study the mechanical response, energy absorption, and failure modes of AFS under low-velocity impact. In recent years, a great deal of research has been invested in the study of the adhesive-bonded AFS’ impact resistance. Mohan et al. [18] compared the impact response of three different sandwich structures, and the results indicated that the panel material not only affects the energy absorption but also affects the failure mode of the foam core. Zhao et al. [19] analyzed the impact resistance of AFS with mild steel panels and quantitatively evaluated the effects of panel thickness, core height, and density on the structural energy absorption performance. Sun et al. [20] investigated the effect of the gradient of core layer density and different panel materials on low-velocity impact performance, the gradient of core layer density was found to have a significant effect on the deformation and failure of the front panel. Islam et al. [21] studied the low-velocity impact response of closed-cell aluminum foam under the effect of indenters with different shapes, revealing that the mechanical response of aluminum foam under low-velocity impact significantly depends on the indenter shape and initial impact energy. However, the research on the low-velocity impact performance of AFS by domestic and foreign scholars is based on the AFS prepared by the adhesive process. Investigation into the mechanical behavior and energy absorption of AFS with integrated molding, high-quality metallurgical bonding interfaces, and short process preparation under low-velocity impact is still absent.

In this paper, AFS with interfacial metallurgical bonding was successfully prepared via the powder cladding process, being suitable for engineering large-size products. The effects of impact energy, panel thickness, and specimen density on the dynamic mechanical response and failure mode of AFS were systematically investigated by drop hammer impact test. The typical failure modes of the panel and foam core macroscopic of AFS with metallurgical bonding interfaces under impact loading are investigated. Deformation and damage of the cell at the cell/membrane level are investigated to reveal the energy dissipation mechanism of the sandwich panel. Meanwhile, AFS’s energy absorption characteristics were quantitatively evaluated by using structural energy absorption indicators, which can provide theoretical and technical support for the engineering application of metallurgical bonding of lightweight AFS.

## 2. Materials and Methods

### 2.1. Materials

**Figure 1:**
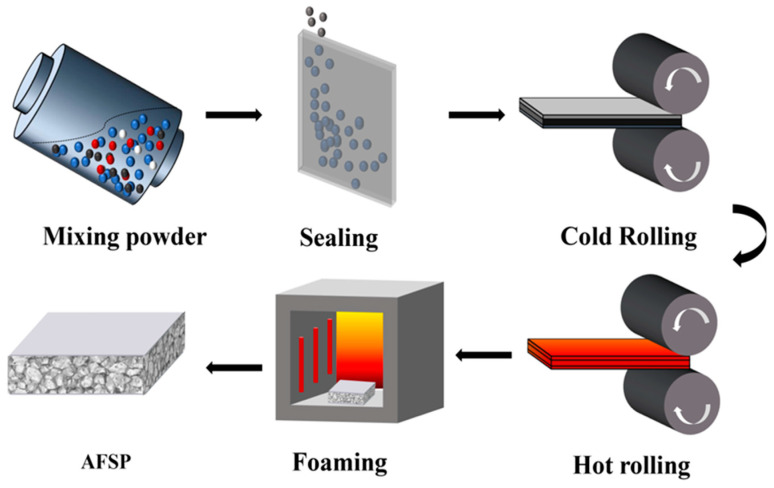
Schematic illustration of the preparation process of AFS.

The schematic illustration of the preparation process of the AFS is shown in Figure 1. In the powder mixing stage, the powders of Al, Si, Cu, Mg, and TiH_2_ (Oxidation treatment at 470 °C for 1.5 h as a foaming agent) are placed in the three-dimensional mixer with an SYH capacity of 5 L. The mixed powder composition and particle size are displayed in Table 1.

The specimen density is calculated by dividing the overall mass of the sandwich panel by its volume. To obtain different density gradients of AFS, the mixing powder stage is loaded with varying masses of mixed powder. Meanwhile, in order to mix various powders evenly and prevent agglomeration, steel mixing balls of Φ5 mm are used in the powder mixing stage. After a preliminary study, it was determined that the best mixing time that could make the powder as homogeneous as possible was 3 h. The mixed powder is sealed in a welded cavity made of 3003 aluminum alloy sheets, and the end of the cavity is filled with asbestos. Furthermore, the cold rolling stage is carried out. The pre-bonding of the powder with the panel is achieved through a multi-pass rolling process with a small rolling reduction on a two-roller mill to exclude as much excess gas as possible between the powder particles. When the cold rolling was completed, hot rolling was performed. The main purpose of hot rolling is to further densify the core layer powder and thin the panel to the required thickness.

The DK7745 wire cutter (Taizhou Poussy CNC Machinery Technology Co., Taizhou, China) was used to cut out the low-density areas at the edges of the precursor obtaining a high-density foamable precursor with good expansion properties. Finally, the samples were heated to 620 °C and foamed in the KSL-1100X-L muffle furnace (Hefei Kejing Materials Co., Hefei, China). In addition, a height-limiting mold was used to further ensure the samples’ flatness and obtain the desired expansion height.

### 2.2. Low-Velocity Impact Test

**Figure 2:**
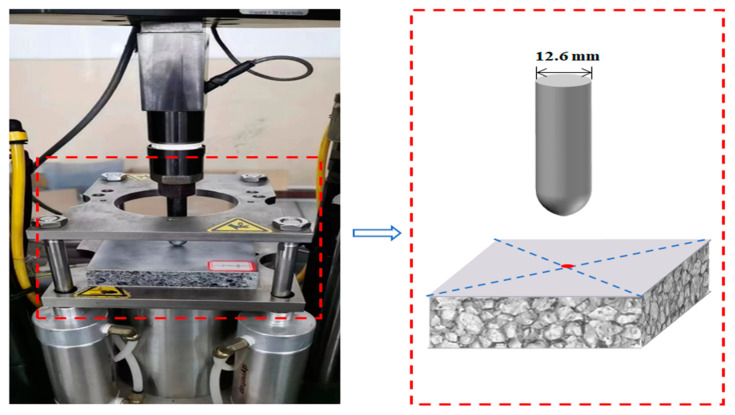
INSTRON 9250HV drop hammer impact tester.

The INSTRON 9250 HV drop hammer impact tester (Norwood, MA, USA) used in this paper is shown in Figure 2. The drop hammer tester is equipped with a double-layer pneumatic clamp and has a square shape with a circular area of 75 mm diameter in the middle. During the experiment, the crosshead used to fix the drop hammer is released and falls freely along the two guide frames in a vertical direction to impact the specimen in a circular area. During the impact test, the sensor automatically collects data relating to changes in load, displacement, and time. Meanwhile, the force-displacement curve is integrated by a computer data acquisition system to obtain the change in impact energy absorbed by the specimen. For this test, a hemispherical indenter with a diameter of 12.6 mm and a mass of 12.250 kg was selected for the test machine. The test sample size of 100 × 100 mm^2^, AFS in the low-velocity impact of the experimental design is summarized in Table 2. To reduce the duplication of experiments, a group of samples was selected as a comparison sample. The mechanical response of the AFS was compared by Group 1 and the comparison sample for different impact energy, other parameters such as panel thickness, specimen density, and core thickness were the same. Similarly, Group 2 and comparison samples were compared for three different panel thicknesses. Furthermore, Group 3 and comparison samples were compared for three different specimen densities. The initial impact energy of 32 J, 60 J, 90 J, and 120 J was obtained by adjusting the height of the indenter. All the tests on foam materials were repeated three times. The expression of the impact energy was:

(1) U=mgh

where *U* is the impact energy, *m* is the drop indenter’s mass, *g* is the acceleration of gravity, and *h* is the height of the indenter.

To quantitatively assess the energy absorption of AFS, three energy absorption indicators are Peak Load, Energy Absorption (EA), and Specific Energy Absorption (SEA). Peak Load is an important indicator to assess the impact strength of sandwich structures [23] and can be obtained directly from the first peak of the load-displacement curve. EA is the energy absorption value of the sample during the impact, which can be obtained from the energy-time curve. In the energy-time curve, when the energy absorption value of the sample does not vary with time and remains constant, this constant value is the EA value of the sample. The energy absorption increases steadily with time and finally remains constant, which represents the total energy absorption permanently absorbed by the sandwich panel at the end of the impact event [9]. SEA represents the energy absorption per unit mass and is used to assess the energy absorption efficiency of AFS. It can be expressed as:

(2) $$SEA=\frac{EA}{m}$$

where *m* indicates the sample mass of the AFS.

## 3. Results and Discussion

### 3.1. Microstructure Characterization of Metallurgical Bonding Interfaces

**Figure 3:**
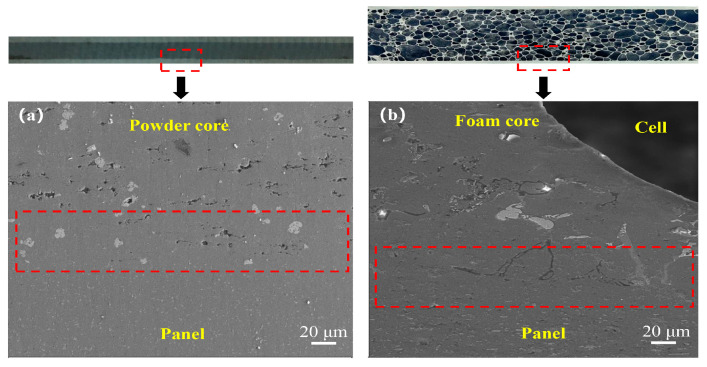
Micro metallurgical interface bonding morphologies: (**a**) Metallurgical bonding interface of the foamable precursor; (**b**) Bonding interface after foaming.

The bonding interface between the panel and the foam core can significantly affect the mechanical properties of AFS [24]. Figure 3a demonstrates the SEM morphology of the microscopic bonding interface of the foamable precursor. It can be observed that a deep fusion between the panel and the core layer powder is obviously achieved through the powder cladding rolling process. Meanwhile, the bonding interface layer (marked with a red dotted line) was without any physical interruptions such as cracks or air gaps. Zhang et al. [25] analyzed the deformation characteristics of AFS during the rolling process, and the results show that the foamable precursor forms a good metallurgical bonding between the panel and the powder core under the action of rolling force. The bonding mechanism is that the panel exposes a fresh metal surface under the rolling force and the powder particles near the panel fill the metal surface, thus bonding tightly with the metal panel.

Figure 3b displays the metallurgical bonding interface (marked with a red dotted line) of AFS after foaming. Compared with Figure 3a, a large number of monolithic powder particles were alloyed at high temperatures. Huang et al. [13] studied the microstructure of this composition, and the results exhibited that the core layer aluminum matrix is mainly composed of alloying phases such as Mg_2_Si, Al_2_Cu, eutectic silicon, and Al_4_Cu_2_Mg_8_Si_7_. Moreover, the diffusion of elements such as Al, Si, Mg, and Cu from the core layer to the panel occurred.

### 3.2. Mechanical Response to Different Impact Energy

Impact energy is one of the critical influencing elements of the dynamic response of sandwich structures, revealing the mechanism of structural damage. The mechanical response and damage modes of aluminum foam sandwich panels under four different impact energy were investigated by comparing Group 1 and CS. The impact energy of 32 J, 60 J, 90 J, and 120 J corresponded to initial impact velocities of 2.28 m/s, 3.13 m/s, 3.83 m/s, and 4.43 m/s, respectively.

**Figure 4:**
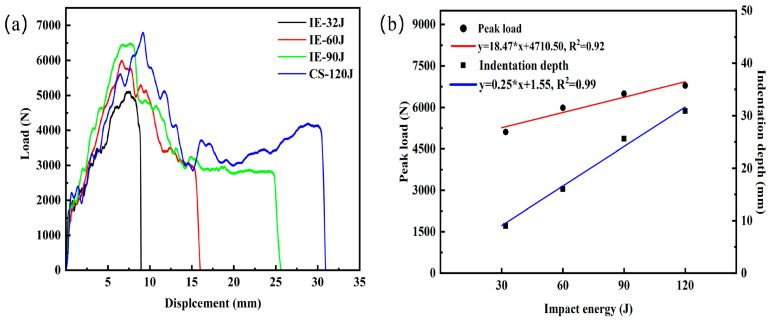
Mechanical response at different impact energy: (**a**) Load-displacement curves; (**b**) Linear fit of peak load and the indentation depth at different energy.

Figure 4a shows the measured load-displacement curves of the AFS at different impact energy. It can be observed that as the impact energy increases from 32 J to 120 J, the corresponding peak loads increase by 17.20%, 8.45%, and 4.51%, while the indentation depths increase by 77.78%, 60%, and 20.70%, respectively. The linear fit of peak load and the indentation depth at different energy are shown in Figure 4b, where it can be visualized that both peak load and indentation depth increase linearly with impact energy.

**Figure 5:**
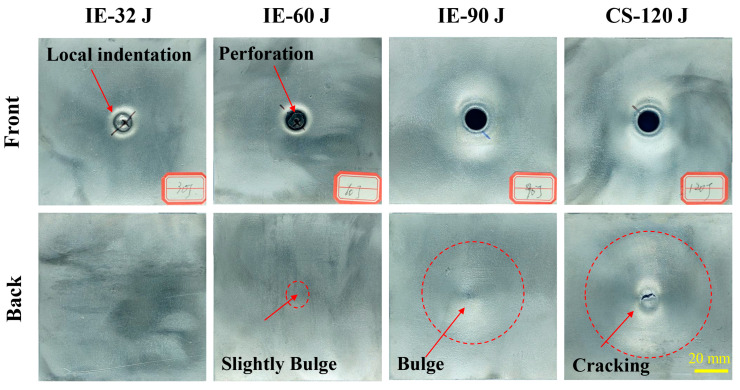
Failure modes of panels of AFS under different impact energy.

**Figure 6:**
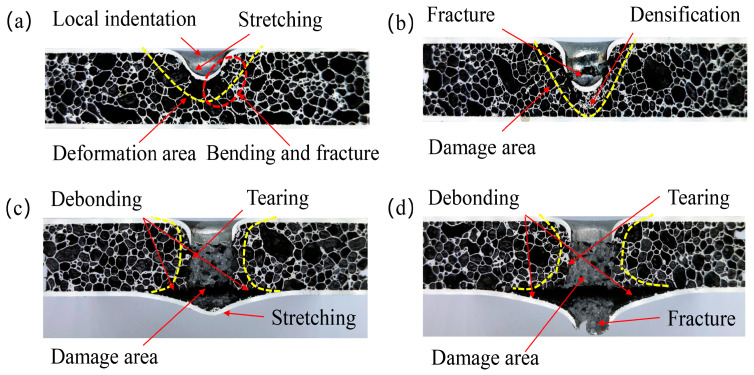
The failure mode of the AFS core layer under different impact energy: (**a**) 32 J; (**b**) 60 J; (**c**) 90 J; (**d**) 120 J.

Figure 5 reveals the failure modes of the panels of the AFS under different impact energy. The failure modes of the core layer of the AFS under different impact energy are shown in Figure 6. When the impact energy is 32 J, it can be seen from the load-displacement curve (see Figure 4a) that the load value increases nonlinearly with the increase in displacement. The phenomenon is because the equivalent contact area between the punch and the AFS increases in a non-linear manner during the impact process. The load value experienced a dramatic decline after reaching its peak, which indicates that the punch rebounded. Meanwhile, as can be seen from the impact energy of 32 J in Figure 5, the spherical punch produces only local indentations in the front panel, showing ductile damage. What is more, neither the front nor the back was fractured or cracked. In combination with the stretching produced by the plastic deformation of the aluminum panel observed in Figure 6a and the bending and fracture of the cell wall (marked by the red oval dashed box) below the local indentation, indicating that the core layer absorbs part of the impact energy.

When the impact energy was 60 J, the load value did not drop abruptly immediately after reaching the peak. However, the load-displacement curve showed a short plateau until the end of the plateau stage. From IE-60 J in Figure 5, it can be observed that with the increase in impact energy, the plastic deformation of the front panel is not enough to dissipate all the impact energy to be completely penetrated by the punch. The front panel has severe local indentation and damage. At the same time, it can be observed in Figure 6b that below the fractured front panel in the damaged area appears obvious cell compression and densification. Moreover, the back panel exhibits a slight bulge indicating that the co-action of compression and shear of the foam aluminum core layer pore further absorbs the remaining impact energy. Thus, the load-displacement curve goes through a short plateau stage.

When the impact energy is 90 J, the load-displacement curve undergoes a more prolonged plateau stage for the load value after the peak load is reached. The punch perforated the front panel but did not perforate the back panel, as shown in Figure 5. Meanwhile, as shown in Figure 6c, between the back panel and the foam core showed a significant tearing and local delamination phenomenon, indicating that most of the impact energy is absorbed by the sandwich structure. In addition, part of the energy is absorbed by the tearing of the cell between the foam core and the back panel.

When the impact energy is 120 J, the load-displacement curve can be divided into three distinct areas based on two peak points. Meanwhile, the front panel and foam core are completely penetrated under 120 J impact energy (see Figure 5 and Figure 6d). Compared with IE-90 J producing cracking, the damage range of the back panel is further expanded. The increase in the load value after the plateau stage is due to the combined effect of the cell compression and shearing to play a buffering role against the movement of the punch, and the load value thus increases with the indentation depth [26]. The radial cracking of the back panel indicates that the impact load is gradually transferred to the back panel when the core layer of the cell is progressively compressed to complete densification by the punch. However, it can be seen that the punch does not entirely penetrate the back panel and the laminated structure still has some protective effect.

Comparing the plateau stages produced by impact velocities of 3.13 m/s, 3.83 m/s, and 4.43 m/s, respectively. It was found that an obvious hardening phenomenon occurred with the increase in impact velocity during the plateau stages. With increasing impact velocity, the plateau stage loads tend to increase significantly. The reason for this may be due to the apparent strain rate sensitivity of low-density aluminum foams. Sahu et al. [27] studied the low-velocity impact indentation rate sensitivity of aluminum foam with different densities, and the study showed that densities below 0.53 g.cm^3^ have higher velocity sensitivity. The core density of the Group 1 sample can be calculated by subtracting the mass of the front and back panels to give a density of 0.36 g.cm^−3^, which is a low-density aluminum foam. Meanwhile, it is worth noting that unlike the debonding failure of AFS prepared by adhesive, sandwich panels with metallurgical bonding interfaces, which cause back panel failure, are delaminated due to the tearing of the back panel and the cell. The large number of torn aluminum foam cells remaining in the back panel is observed in Figure 6d, which indicates that the interfacial bond strength is much higher than the core strength.

**Figure 7:**
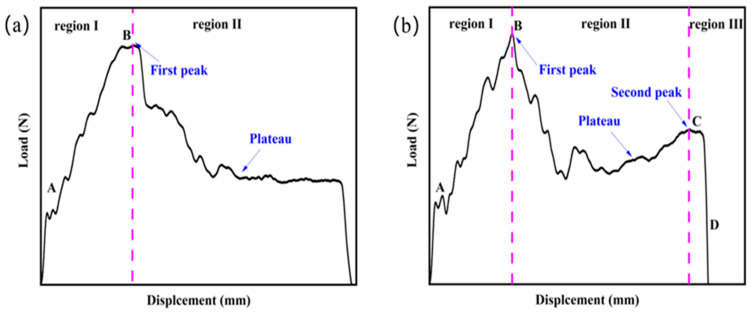
Typical load-displacement curves: (**a**) Mode A; (**b**) Mode B.

The load-displacement curves under different impact energy can be divided into two modes, with and without the plateau stage. The latter can further identify two types of typical load-displacement curves: Mode A and Mode B, as shown in Figure 7. Mode A has a single-peak curve, and Mode B has a double-peak curve. The load-displacement curve of Figure 7a is divided into region Ⅰ and region Ⅱ according to peak point B. Mode B shows two peak points, so the load-displacement curve is divided into region Ⅰ, region Ⅱ, and region Ⅲ.

Failure analysis of the panel and foam core shows that wrinkling and yielding of the front panel occur mainly in region Ⅰ, while no change occurs in the back panel. Meanwhile, the point A in the curve indicates a slight plastic deformation of the front panel without being penetrated. Region Ⅱ mainly occurs in the penetration of the front panel, as well as the compression and shear failure of the foam core and the bulging failure of the back panel. Region III undergoes shear failure of the core layer as well as cracking of the back panel, where the appearance of peak points B and C marks the failure of the front and back panels, respectively. In addition, Point D indicates that the entire specimen has been completely penetrated. Model B load-displacement curve exhibits three stage characteristics typical of the penetration model.

**Figure 8:**
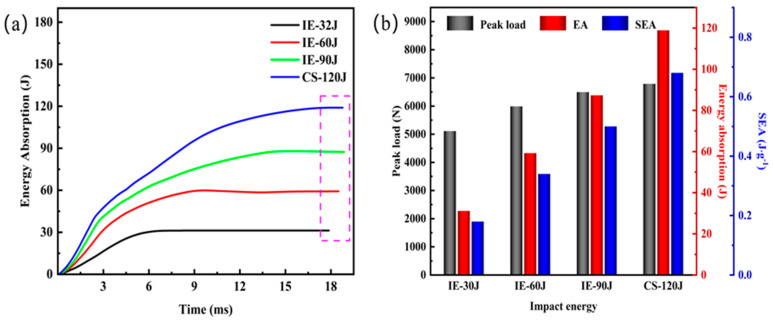
Evaluation of energy absorption performance at different impact energy: (**a**) Energy-time curves; (**b**) Comparison of energy absorption index of the structure.

Figure 8a shows the energy time curves under different impact energy. The boxes (marked with purple dashed lines) in the figure are the EA values of AFS under four different impact energy. The values can be calculated by integration through the curves surrounded by force and displacement, and the impact process’s energy absorption can be output by the drop hammer testing machine. It can be seen that as the impact energy increases, the EA values of the samples are 31.15 J, 59.24 J, 87.35 J, and 118.9 J, which account for 97%, 99%, 97%, and 99% of the total impact energy, respectively. The above results indicate that the AFS has excellent energy absorption efficiency under low-velocity impact.

Figure 8b reveals the comparison of Peak Load, Energy Absorption, and Specific Energy Absorption indexes under different impact energy. It can be observed that all three indexes increase linearly with the impact energy, indicating that the AFS has a better impact energy absorption effect. It also can be analyzed that the panel thickness of 1.7 mm and core density of 0.7 g.cm^−3^ still have a high energy absorption efficiency under 120 J penetration energy.

### 3.3. Mechanical Response of Different Structural Parameters at the Same Impact Energy

To exert the maximum energy absorption and energy absorption efficiency of AFS, the impact energy of 120 J was used to drop hammer impact experiments on AFS with different panel thicknesses of 1.3 mm, 1.7 mm, and 2.1 mm and three different densities of 0.6 g.cm^−3^, 0.7g.cm^−3^, 0.8 g.cm^−3^, respectively.

#### 3.3.1. Influence of Panel Thickness

**Figure 9:**
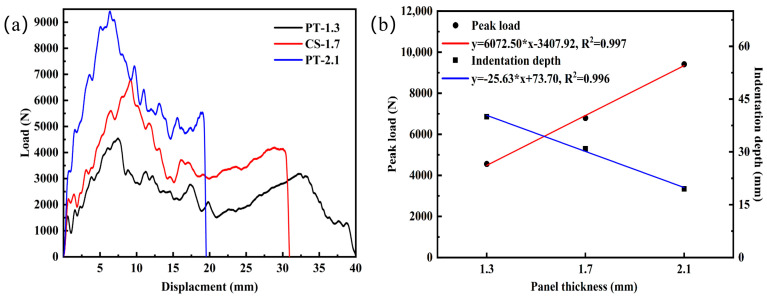
Mechanical response at different panel thicknesses: (**a**) Load-displacement curves; (**b**) Linear fitting diagram of peak load and indentation depth under different panel thicknesses.

Figure 9a shows the load-displacement curves for different panel thicknesses, and it can be seen that the panel thickness has an essential effect on the load-displacement curve. The density of the foam core is 0.36 g.cm^−3^ for all three different panel thicknesses. As the panel thickness increases from 1.3 mm to 2.1 mm, the corresponding peak loads increase by 49% and 68% while the indentation depth decreases by 23% and 37%, respectively. The results show that the impact strength of the structure increases as the panel thickness increases. Figure 9b visually depicts the variation of peak load and indentation depth with panel thickness by linear fitting. The peak load increases correspondingly with panel thickness in a positive correlation, while the indentation depth decreases with panel thickness in a negative correlation.

**Figure 10:**
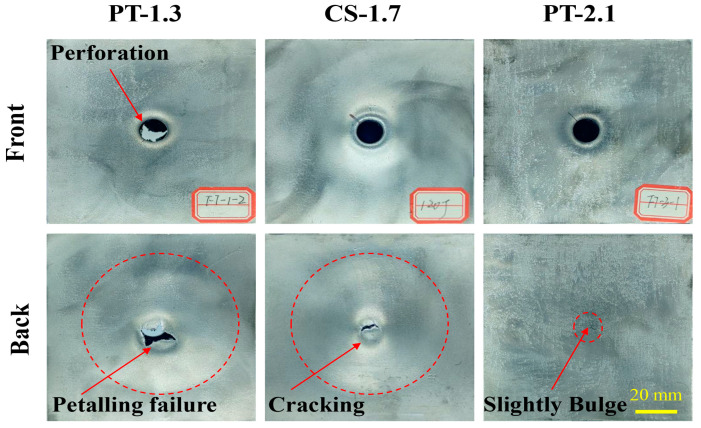
Failure modes of panels of AFS with different panel thicknesses.

Figure 10 shows the failure modes of the panels with different panel thicknesses. When the panel thickness is 1.3 mm after the punch penetrates the front panel and core layer, the impact load is gradually transferred to the back panel. After the punch penetrates the back panel, the back panel has a pedaling failure, and the aluminum foam fragments clogged in the damaged area are entirely removed. As the panel thickness increases to 1.7 mm, the protective effect of the panel is gradually enhanced, and the back panel is transformed from a petal-shaped tear to a radial crack that still has a certain degree of protection. At a panel thickness of 2.1 mm, the back panel produced only a less obvious bulge, which can be described as ductile-brittle damage.

**Figure 11:**
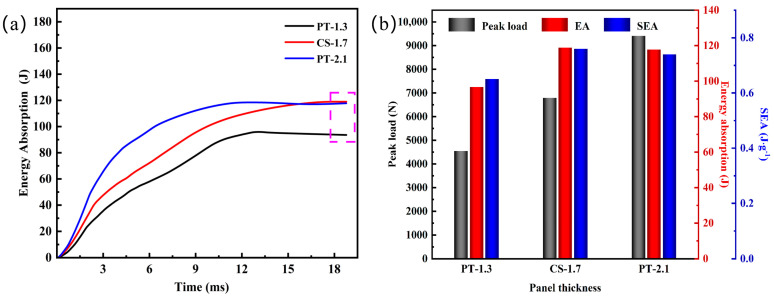
Evaluation of energy absorption performance at different panel thicknesses: (**a**) Energy-time curves; (**b**) A comparison of the energy absorption index of the structure.

Figure 11a shows the energy-time curves. The constant value marked by the purple dashed line in the curve is the EA value. The EA values of the samples are 96.73 J, 118.9 J, and 117.78 J, accounting for 81%, 99%, and 98% of the total impact energy, indicating that the energy absorption of the sandwich panel is enhanced with the increase in the panel thickness.

It can be seen in Figure 11b that with the increase in panel thickness, the peak load increases linearly, and the impact resistance of the structure increases continuously. EA and SEA increase correspondingly when the panel thickness increases from 1.3 mm to 1.7 mm, but with the increase in panel thickness to 2.1 mm, EA and SEA decrease instead. This is because when the panel thickness increases to a certain degree, the plastic deformation capacity of the panel is reduced, which hinders the transfer of impact load to the panel. Thus, the energy absorption EA is reduced, and the corresponding energy absorbed per unit mass is reduced. Therefore, an excellent panel thickness selection is more favorable for designing high-impact aluminum foam sandwich panels. In summary, panel thickness significantly affects the impact resistance of aluminum foam sandwich panels and the failure mode of the back panel.

#### 3.3.2. Influence of Sample Density

**Figure 12:**
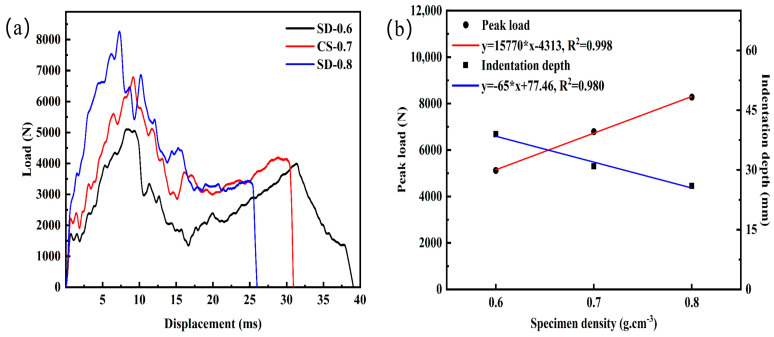
Mechanical response at different densities: (**a**) Load-displacement curves; (**b**) Linear fitting plots of peak load, and the indentation depth at different densities.

Figure 12a shows the load-displacement curves of AFS at different densities. The panel thickness used for the three different density specimens was 1.7 mm. As the density increases from 0.6 g.cm^−3^ to 0.8 g.cm^−3^, the corresponding peak loads increase by 33% and 22%, and the indentation depths decrease by 21% and 16%, respectively. The above results show that the impact resistance of the structure is effectively enhanced as the density of AFS increases.

Figure 12b exhibits a linear fit to visualize the variation of peak load and indentation depth with panel thickness. The peak load increases correspondingly with sample density in a positive correlation, while the indentation depth decreases with sample density in a negative correlation. Meanwhile, it is noteworthy that the plateau stage of the load-displacement curve of the low-density AFS shows an obvious valley, while the plateau areas of the density of 0.7 g.cm^−3^ and 0.8 g.cm^−3^ are relatively stable.

This can be explained by the fact that the low-density-AFS has a high volume fraction of the gas phase, and the cell’s shape is mainly polygonal, with many weak connections between the cells [27]. Therefore, the load value is sharply reduced to the valley value under impact loading, showing a clear brittle behavior. However, with a large amount of aluminum foam being compressed and the wall being torn by the punch, the accumulation of these internal defects gradually dominates. As a result, the load-displacement curve shows the load value increasing with the indentation displacement until the impact load is transferred to the back panel. The impact load transfer pattern indicates that the density has a hysteresis and hindrance effect on the impact load, and compared with the high-density aluminum foam sandwich panel, it is obvious that the stability of the plateau area deformation is worse.

**Figure 13:**
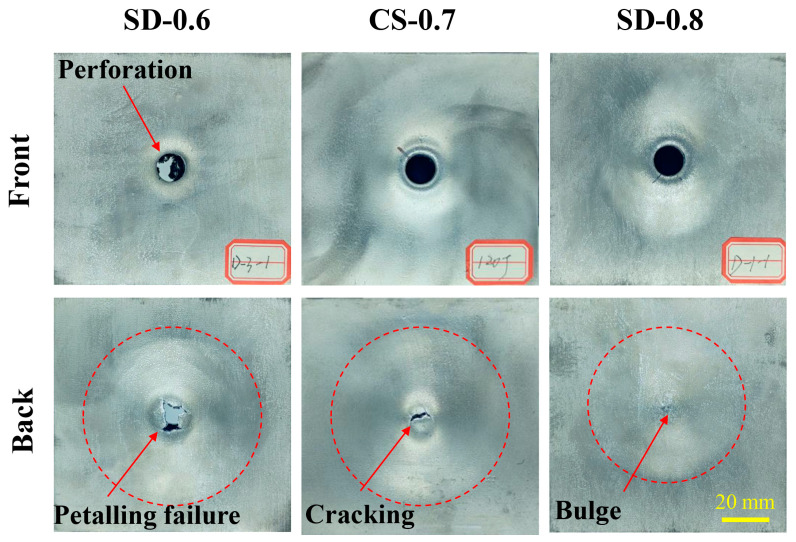
The failure mode of the panels of AFS with different densities.

Figure 13 shows the failure mode of the panels of AFS with different densities. It can be seen that the front and back panels have similar failure modes as in Figure 10. The front panels are penetrated under the impact load. The difference is that the damaged area of the back panel of AFS with a density of 0.8 g.cm^−3^ is large. The bulge failure is more obvious, which indicates that the density has a vital role in the structural load-bearing energy.

**Figure 14:**
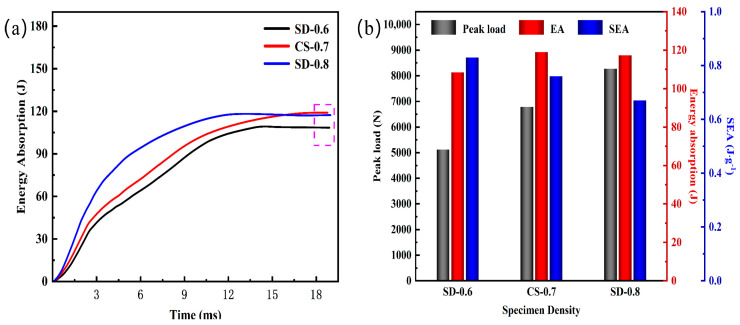
Evaluation of energy absorption performance at different densities: (**a**) Energy-time curves; (**b**) Comparison of structural energy absorption indicators.

Figure 14a shows the energy-time curves. The constant value marked by the purple dashed line in the curve is the EA value. The EA values of the samples are 108.43 J, 118.9 J, and 117.30 J, accounting for 91%, 99%, and 98% of the total impact energy, indicating that the energy absorption of the sandwich panel is enhanced with the increase in density.

It can be observed in Figure 14b that with the increase in density and decrease in porosity, the peak load will show an increasing trend. The EA value increased from 0.6 g.cm^−3^ to 0.7 g.cm^−3^ with the corresponding increase in density. Still, when the density continued to increase to 0.8 g.cm^−3^, the EA value no longer increased but decreased, combined with the trend of reducing SEA, indicating that the increase in excessive density increased the mass of the specimen while lowering the energy absorption capacity of the sandwich panel. The density of AFS is achieved by changing the powder mass in the cavity during the sealing stage. The high density of AFS represents a higher powder filling capacity, so in engineering applications, it is necessary to select the density of AFS by energy absorption efficiency, cost, and other factors.

## 4. Conclusions

In this study, AFS with metallurgical bonding interfaces was successfully prepared by the powder cladding rolling method. By testing different impact energy, the load-bearing capacity, energy absorption, and failure modes of AFS were systematically explored, and the influence law of panel thickness and density on the impact resistance of the structure was investigated based on the maximum energy absorption efficiency of the structure, and the obtained conclusions are as follows:

(1) The physical interruptions, such as cracks or holes were not observed in the interfacial bonding area of the AFS prepared by the powder cladding rolling method, and an excellent metallurgical bond has been achieved.
(2) AFS has two typical load-displacement curves under different impact energy: a single peak curve under low-impact energy and a double peak curve under high-impact energy. The double peak curve shows prominent three-stage characteristics, which dissipates impact energy in the first stage mainly by yielding of the front panel; in the second stage, impact energy was dissipated by compression and shear failure of the foam core and tearing of the surrounding cell wall; in the third stage, impact energy was dissipated mainly by the shear failure of the foam structure and fracture of the back panel.
(3) With the increase in thickness of the panel, the structure’s impact strength was gradually enhanced. Furthermore, the increase in panel thickness above 1.7 mm would reduce the plastic deformation capacity of the panel and result in a reduction in the energy absorption efficiency of the structure.
(4) The increase in density can improve the impact strength of the sandwich structure, but it is inversely proportional to its impact energy absorption capacity. The force-displacement curves for sample density above 0.6 g.cm^−3^ have relatively higher deformation stability and less fluctuation in the plateau region compared to AFS with a specimen density of 0.6 g.cm^−3^.

## Figures and Tables

**Table 1:** Mixed powder composition and particle size [22].

Composition	Range Size (μm)	Purity (%)	Content
Al	<45	99.70	85%
Si	<38	99.50	6%
Mg	<75	99.90	4%
Cu	<38	99.90	4%
TiH_2_	<45	99.70	1%

**Table 2:** Experimental design of low-velocity impact of AFS.

	Specimen Code	Panel Thickness (mm)	Height of Core (mm)	Specimen Density (g.cm^−3^)	Impact Energy (J)
Comparison samples	CS	1.7	18.5	0.7	120
Group 1	IE-32J	1.7	18.5	0.7	32
	IE-60J	1.7	18.5	0.7	60
	IE-90J	1.7	18.5	0.7	90
Group 2	PT-1.4	1.3	18.5	0.7	120
	PT-2.1	2.1	18.5	0.7	120
Group 3	SD-0.6	1.7	18.5	0.6	120
	SD-0.8	1.7	18.5	0.8	120

CS: Comparison samples, IE: Impact energy, PT: Panel thickness, SD: Specimen Density. Attention: CS is used to compare three parameters with different impact energy, panel thickness, and specimen Density.

## Data Availability

Not applicable.
